# Plant latent defense response to microbial non-pathogenic factors antagonizes compatibility

**DOI:** 10.1093/nsr/nwac109

**Published:** 2022-06-10

**Authors:** Yu Yang, Shenglan Chen, Xiaoxuan Wu, Li Peng, Juan I Vílchez, Richa Kaushal, Xiaomin Liu, Sunil K Singh, Danxia He, Fengtong Yuan, Suhui Lv, Rafael J L Morcillo, Wei Wang, Weichang Huang, Mingguang Lei, Jian-Kang Zhu, Paul W Paré, Huiming Zhang

**Affiliations:** Shanghai Center for Plant Stress Biology, Center for Excellence in Molecular Plant Sciences, Chinese Academy of Sciences, Shanghai 201602, China; Shanghai Center for Plant Stress Biology, Center for Excellence in Molecular Plant Sciences, Chinese Academy of Sciences, Shanghai 201602, China; University of Chinese Academy of Sciences, Beijing 100049, China; Shanghai Center for Plant Stress Biology, Center for Excellence in Molecular Plant Sciences, Chinese Academy of Sciences, Shanghai 201602, China; University of Chinese Academy of Sciences, Beijing 100049, China; Shanghai Center for Plant Stress Biology, Center for Excellence in Molecular Plant Sciences, Chinese Academy of Sciences, Shanghai 201602, China; Shanghai Center for Plant Stress Biology, Center for Excellence in Molecular Plant Sciences, Chinese Academy of Sciences, Shanghai 201602, China; Shanghai Center for Plant Stress Biology, Center for Excellence in Molecular Plant Sciences, Chinese Academy of Sciences, Shanghai 201602, China; Shanghai Center for Plant Stress Biology, Center for Excellence in Molecular Plant Sciences, Chinese Academy of Sciences, Shanghai 201602, China; Shanghai Center for Plant Stress Biology, Center for Excellence in Molecular Plant Sciences, Chinese Academy of Sciences, Shanghai 201602, China; Shanghai Center for Plant Stress Biology, Center for Excellence in Molecular Plant Sciences, Chinese Academy of Sciences, Shanghai 201602, China; University of Chinese Academy of Sciences, Beijing 100049, China; Shanghai Center for Plant Stress Biology, Center for Excellence in Molecular Plant Sciences, Chinese Academy of Sciences, Shanghai 201602, China; University of Chinese Academy of Sciences, Beijing 100049, China; Shanghai Center for Plant Stress Biology, Center for Excellence in Molecular Plant Sciences, Chinese Academy of Sciences, Shanghai 201602, China; University of Chinese Academy of Sciences, Beijing 100049, China; Shanghai Center for Plant Stress Biology, Center for Excellence in Molecular Plant Sciences, Chinese Academy of Sciences, Shanghai 201602, China; Shanghai Chenshan Botanical Garden, Shanghai 201602, China; Shanghai Chenshan Botanical Garden, Shanghai 201602, China; Shanghai Center for Plant Stress Biology, Center for Excellence in Molecular Plant Sciences, Chinese Academy of Sciences, Shanghai 201602, China; Shanghai Center for Plant Stress Biology, Center for Excellence in Molecular Plant Sciences, Chinese Academy of Sciences, Shanghai 201602, China; Department of Chemistry and Biochemistry, Texas Tech University, Lubbock, TX 79409, USA; Shanghai Center for Plant Stress Biology, Center for Excellence in Molecular Plant Sciences, Chinese Academy of Sciences, Shanghai 201602, China

**Keywords:** microbial non-pathogenic factors, latent defense response, compatibility, plant-beneficial bacteria, chloroplastic lipid biosynthesis

## Abstract

Unlike microbe-associated molecular patterns (MAMPs) that are readily targeted by host immunity, microbial non-pathogenic factors (NPFs) appear negligible as they do not elicit defense. Little is known about whether and how NPFs may be monitored by hosts to control compatibility. Herein, a forward genetic screening isolated an Arabidopsis mutant with a loss of plant-rhizobacteria mutualism, leading to the disclosure of a plant latent defense response (LDR) to NPFs. The activation of LDR in the mutant, named *rol1* for *regulator of LDR 1*, is triggered by several non-pathogenic volatile organic compounds and antagonizes plant compatibility with the beneficial bacterium *Bacillus amyloliquefaciens* GB03. The activation of LDR in *rol1* is mediated through the prokaryotic pathway of chloroplastic lipid biosynthesis. The *rol1* root microbiome showed a reduced proportion of the *Bacillaceae* family. We propose that, parallel to the forefront immunity to MAMPs, LDR to certain NPFs provides a hidden layer of defense for controlling compatibility with commensal or beneficial microbes.

## Teaser

Microbial non-pathogenic factors (NPFs) conditionally activate plant latent defense response (LDR) that antagonizes compatibility with beneficial bacteria.

## INTRODUCTION

Plants are naturally surrounded by a complex array of microbe-secreted molecules, among which microbe-associated molecular patterns (MAMPs) readily elicit plant immune responses that limit microbe proliferation [1]. Meanwhile, many other microbial metabolites are non-pathogenic factors (NPFs) that seemingly do not elicit host defense. Plant compatibility with commensal or beneficial microbes requires either that the conserved MAMPs evade host recognition [2], or that the MAMP-elicited defense is suppressed [3]; whereas the apparent inertness of plants in mounting a defense response to various NPFs is an often-taken-for-granted assumption. Little is known about whether and how NPFs may be monitored by plants for controlling compatibility.

## RESULTS

**Figure 1.**
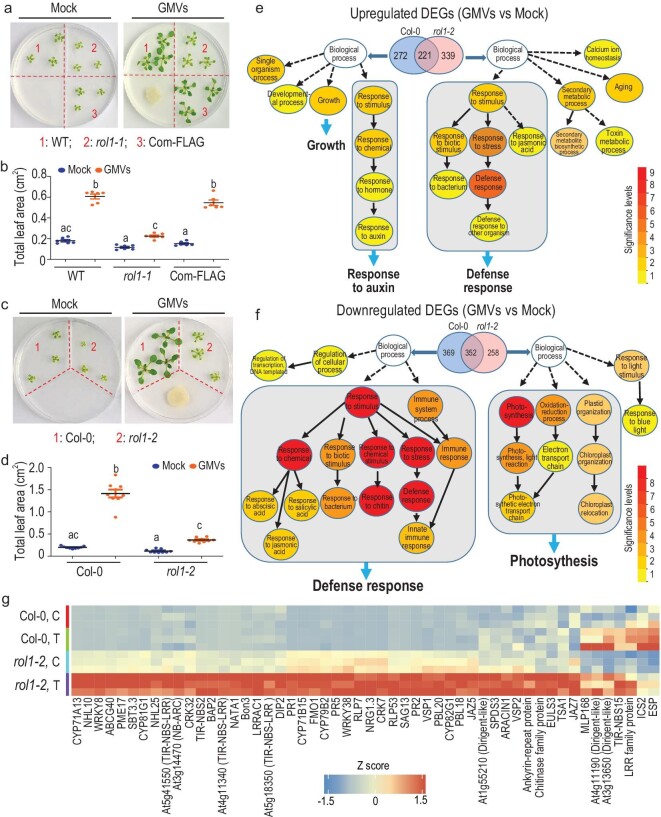
The loss of plant-rhizobacteria mutualism in *rol1* mutants disclosed plant LDR. (a) Compared to its wild type (WT), the EMS mutant allele *rol1-1* showed impaired plant growth promotion, which was restored by the *ROL1* gene complementation (Com-FLAG). The petri dishes contained plastic partitions (dotted lines), which separated the medium for plant growth and the medium for bacteria, so that the bacteria could affect the plant only through volatile emissions. Images were taken 9 days after treatment (DAT). (b) Quantification of plant growth promotion shown in panel (a). Values are mean ± SE, *n* = 6 biological replicates. Different letters denote *P* ≤ 0.05 (Tukey's multiple comparison test). (c) The T-DNA insertion mutant allele *rol1-2* showed impaired growth promotion compared to its wild-type plants (Col-0). Plants were 9 DAT. (d) Quantification of plant growth promotion shown in panel (c). Mean ± SE, *n* = 9 biological replicates. (e and f) Comparative gene ontology (GO) analysis of Arabidopsis genes that were (e) upregulated or (f) downregulated at 2 DAT by GMVs. The Venn diagrams show the numbers of DEGs (differentially regulated genes) identified in Col-0 and *rol1-2*. GO pathways are based on AgriGO V2 (http://systemsbiology.cau.edu.cn/). The color key indicates the significance levels, in which level 9 means the most significant according to the *P* value of the enrichment. Detailed DEG lists and GO terms are provided in Supplementary Data Set S1. (g) GMVs induced (fold changes ≥ 2, BH < 0.05) a group of 52 defense-related genes in *rol1-2* but not Col-0 plants. DEGs shown in the heat map were identified by RNAseq.

*Bacillus amyloliquefaciens* strain GB03 is a beneficial rhizobacterium capable of promoting plant growth [4]. GB03-produced microbial volatiles (GMVs) trigger beneficial effects such as enhanced development of lateral roots and an increase in photosynthetic apparatus [5,6]. In a forward genetic screening of *Arabidopsis thaliana* Ethylmethane sulfonate (EMS) mutants, we isolated a mutant (named later as *rol1-1* for *regulator of latent defense response 1–1*) showing defective growth promotion triggered by GMVs or by GB03 root inoculation (Fig. 1a and b; Fig. S1a and b). Map-based cloning identified a recessive mutation in At2g43710 (Fig. S1c), which was confirmed by gene complementation and a T-DNA insertion allele (Fig. 1a–d; Fig. S1d and f, Fig. S2a and b). *ROL1* encodes a stearoyl-ACP desaturase known as FAB2/SSI2 that converts stearic acid (18 : 0) to oleic acid (18 : 1) [7,8]. Exogenous glycerol mimics *ssi2* mutation in decreasing 18 : 1 levels and in increasing levels of nitric oxide (NO), an important regulator of plant development and stress response [9,10]. Consistently, wild-type plants treated with glycerol or the NO donor S-nitrosoglutathione mimicked *rol1* mutants in showing defective growth promotion (Fig. S2c–f), further confirming the importance of ROL1 for GMV-triggered growth promotion.

**Figure 2.**
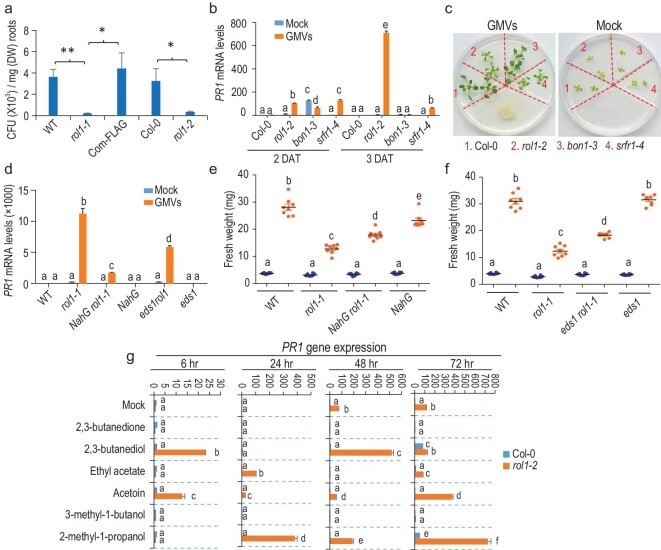
LDR to GMVs antagonizes plant compatibility with GB03. (a) ROL1 dysfunction impairs the root colonization of GB03; mean ± SD, *n* = 4 technical replicates. Three independent experiments were performed with similar results. Student's t-test *P* < 0.05 (^*^) or 0.01 (^**^). (b) GMVs elicited LDR in *rol1-2* and *srfr1-4*, but not *bon1-3*. Quantitative RT-PCR; mean ± SD, *n* = 3 technical replicates. All results of qRT-PCR were confirmed by three independent experiments. Different letters denote significant differences at *P* < 0.05, Tukey's multiple comparison test. (c) The *rol1-2* and *srfr1-4* mutants showed stronger impairments of plant growth promotion than *bon1-3*. Images were taken at 10 DAT. (d) Defects in SA accumulation (*NahG rol1-1*) or signaling (*eds1 rol1-1*) partially suppressed GMV-elicited LDR in *rol1-1*. qRT-PCR; mean ± SD, *n* = 3 technical replicates. (e) Transgenic expression of *NahG* in *rol1-1* partially restored plant growth promotion. Mean ± SE, *n* = 8 biological replicates. (f) A null mutation of *EDS1* in *rol1-1* partially restored plant growth promotion. Mean ± SE, *n* = 8 biological replicates. (g) Several synthetic GMV components induced LDR in *rol1-2* plants. qRT-PCR; mean ± SD, *n* = 3 technical replicates. Values are normalized to the *PR1* expression level in Col-0 mock plants for each time point. The synthetic compounds were applied at dosages that, when the compounds totally evaporate from the agar-containing solid droplets, would yield in volatile concentrations of 32.5 μg (2,3-butanediol), 7.8 μg (2-methyl-1-propanol), 2.5 μg (3-methyl-1-butanol), 6.2 μg (ethyl acetate), 9.7 μg (2,3-butanedione) and 28.5 μg (acetoin) per mL free space in the petri dish, which resembled the ratio among the six GMV components in natural GMVs as previously reported [16]. Different letters denote significant differences at *P* < 0.05, Tukey's multiple comparison test.

We sought to understand why GMVs failed to trigger growth promotion in *rol1* mutants. Transcriptome analysis revealed that, in *rol1-2*, compared to its wild-type plants, GMVs not only failed to induce the growth-related processes but also caused suppression of photosynthesis (Fig. 1e and f). Importantly, while GMVs are non-pathogenic to wild-type plants, the *rol1* mutants responded to GMVs with a strong activation of defense (Fig. 1e–g; Fig. S3a; Supplementary Data Set S1), indicating a ROL1-dependent change in the plant's judgment of GB03. Consistently, root colonization of GB03 was impaired in the *rol1* mutants (Fig. 2a). These results demonstrate that certain NPFs can be perceived by plants for controlling compatibility. To highlight this hidden layer of defense, we called it the latent defense response (LDR) to NPFs.

ROL1 dysfunction causes autoimmunity [8] (Fig. 1g), yet LDR is not necessarily linked with autoimmunity, because GMV-elicited LDR was observed in *srfr1-4* but not *bon1-3* (Fig. 2b; Fig. S3b), which are autoimmune mutants that are defective in a negative transcriptional regulator of effector-triggered immunity and a plasma-membrane-localized protein that suppresses R gene expression, respectively [11,12]. LDR antagonizes growth promotion, as indicated by the stronger impairment of growth promotion in *rol1-2* and *srfr1-4* than in *bon1-3* (Fig. 2c; Fig. S3c). This antagonism was also shown in *rol1-1* carrying the *NahG* transgene or *eds1* mutation, which disrupted the production and signaling of the defense-related phytohormone salicylic acid (SA), respectively [13,14], because LDR was partially reduced while growth promotion was partially restored (Fig. 2d–f; Fig. S4a–c). The remaining LDR in these double mutants is independent of MPK3 and MPK6 (Fig. S4d), two kinases that can mediate SA-independent defense [15].

Among the over 30 compounds of GMVs [16], we examined 6 synthetic main components. LDR was elicited in *rol1-2* by 2-methyl-1-propanol, 2,3-butanediol and acetoin, but not a structurally similar compound 2,3-butanedione (Fig. 2g; Fig. S5a–c), which suppresses microbial induction of reactive oxygen species (ROS) [17]. LDR was not induced by elevated levels of the respiration product carbon dioxide (Fig. S5b and c). Altogether these results demonstrate that certain NPFs can be subject to an LDR that antagonizes plant compatibility with the microbes.

**Figure 3.**
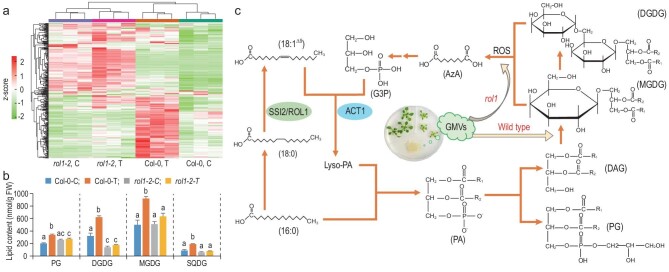
ROL1 dysfunction turns the plant association with GB03 from beneficial into unfavorable. (a) ROL1 dysfunction and GMVs showed contrasting impacts on plant lipidome. The heat map shows 321 species of lipid and fatty acids detected by UPLC-qTOF-MS. Three biological replicates harvested at 3 DAT. (b) GMVs enhanced the production of major chloroplastic lipids in Col-0 but not the *rol1-2* mutant. Different letters denote significant differences at *P* < 0.05, Tukey's multiple comparison test, for each lipid category. (c) The prokaryotic pathway of lipid biosynthesis [19], which starts from ACT1-catalyzed acylation of G3P with 18 : 1, plays a central role in dictating plant compatibility with GB03. In wild-type plants, GMVs enhance the production of chloroplastic lipids in supporting plant growth promotion; whereas in the *rol1* mutants, attempts to enhance chloroplastic lipid production would exacerbate 18 : 1 deficiency, making the association unfavorable for plants. The oxidative cleavage of MGDG and DGDG is included to highlight the negative impact of ROS accumulation on this pathway.

We next sought to understand why the *rol1* mutants activate LDR to GMVs. A total of 321 species of lipid and fatty acids were detected in the plant lipidome (Supplementary Data Set S2), which was substantially altered by either ROL1 dysfunction or GMVs in largely distinct sub-portions (Fig. 3a; Fig. S6a–c). GMVs not only failed to induce similar lipidome changes in *rol1-2* as in the wild-type plants, but also exacerbated the lipidome disruptions caused by ROL1 dysfunction (Fig. 3a), indicating that the association with GB03 was unfavorable for *rol1* mutants. Importantly, the wild-type plants responded to GMVs with significantly increased levels of phosphatidylglycerol (PG), monogalactosyldiacylglycerol (MGDG), diagalactosyldiacylglycerol (DGDG) and sulfoquinovosyldiacylglycerol (SODG) (Fig. 3b), which are the four major categories of chloroplastic lipids [18]. In contrast, the *rol1-2* mutant plants not only already accumulated lower levels of DGDG compared to the untreated wild-type plants, but also failed to show increases in these chloroplastic lipids in response to GMVs (Fig. 3b). On one hand, these results provide a metabolic mechanism for GB03’s beneficial effects in increasing the photosynthetic apparatus in wild-type plants [6], because MGDG and DGDG constitute the bulk of membrane lipids in chloroplasts and are major components of the thylakoid membrane [18]. On the other hand, because MGDG and DGDG are synthesized through the prokaryotic pathway that starts from ACYLTRANSFERASE 1 (ACT1)-catalyzed acylation of glycerol-3-phosphate (G3P) with 18 : 1 [19] (Fig. 3c), the GMV-induced attempts to enhance chloroplastic lipid production would threaten to exacerbate the 18 : 1 deficiency in *rol1*, making the association with GB03 unfavorable for *rol1*; consistently, the *rol1-2* plants responded to GMVs with transcriptional repression of photosynthesis genes instead of elevations in MGDG and DGDG (Fig. 1f; Fig. 3b). Therefore, the prokaryotic pathway of lipid biosynthesis plays a central role in dictating plant compatibility with GB03.

**Figure 4.**
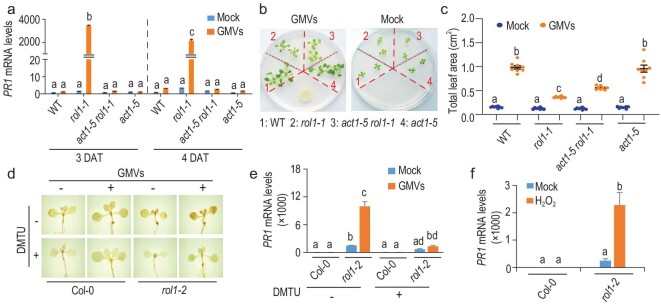
LDR is conditionally activated under unfavorable bacterial association. (a) The *act1-5* mutation completely suppressed LDR in *rol1-1*. qRT-PCR; mean ± SD, *n* = 3 technical replicates. Different letters denote significant differences at *P* < 0.05, Tukey's multiple comparison test. (b) The *act1-5* mutation partially restored plant growth promotion in *rol1-1*. Images were taken at 11 DAT. (c) Quantification of plant growth promotion shown in panel (b). Values are mean ± SE, *n* = 8 biological replicates. (d) GMVs triggered H_2_O_2_ over accumulation in *rol1-2* plants, while the H_2_O_2_ accumulation was abolished by the H_2_O_2_ scavenger dimethylthiourea (DMTU). (e) GMV-triggered LDR in *rol1-2* was blocked by DMTU. Plants were treated with GMVs and DMTU at the same time and harvested at 4 DAT. qRT-PCR; mean ± SD, *n* = 3 technical replicates. (f) Exogenous application of H_2_O_2_ mimicked GMVs in triggering LDR in the *rol1-2* plants. qRT-PCR; mean ± SE, *n* = 3 biological replicates.

Consistent with this notion, LDR in *rol1-1* was completely suppressed by the *act1-5* mutation (Fig. 4a; Fig. S7a), indicating that the ACT1-dependent consumption of 18 : 1 is necessary for GMV-induced LDR. The restoration of growth promotion in *act1-5 rol1-1* was partial (Fig. 4b and c), likely due to the disrupted chloroplastic lipid homeostasis as indicated by leaf chlorosis (Fig. S7b). The levels of H_2_O_2_ and gene expression of PRX34, an apoplastic peroxidase crucial for ROS production [20], were elevated by ROL1 dysfunction and further increased by GMVs (Fig. 4d; Fig. S8a and b), indicating exacerbated oxidative stress that would also make the association with GB03 unfavorable for *rol1* mutants, because ROS-mediated oxidation of MGDG and DGDG produces azelaic acid that primes SA-dependent defense and increases G3P that drives ACT1-dependent consumption of 18 : 1 [21,22] (Fig. 3c). Consistently, the H_2_O_2_ scavenger dimethylthiourea blocked GMV-triggered LDR, whereas exogenous H_2_O_2_ mimicked GMVs in eliciting LDR in *rol1-2* (Fig. 4e and f; Fig. S8c–e), indicating that the activation of LDR in *rol1* plants is mediated through the ROS-dependent perception of GMVs.

**Figure 5.**
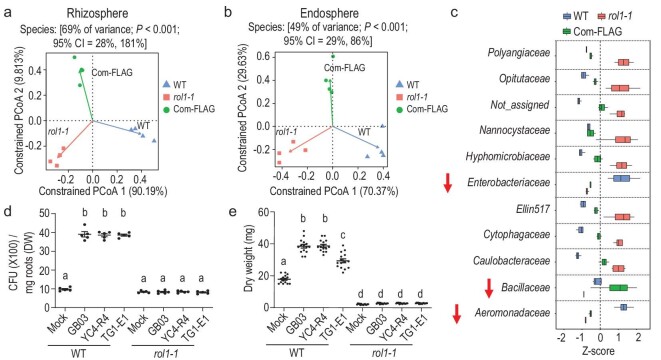
Alterations in natural root microbiome highlight reductions in plant association with *Bacillaceae*. (a) Principal coordination analysis (PCoA) of all Operational Taxonomic Units (OTUs) detected in the rhizosphere compartment of *rol1-1*, the wild-type plants (WT) and the complementation line (Com-FLAG). (b) PCoA of the OTUs within the endosphere compartment. (c) The bacteria families whose rhizosphere enrichment was affected by the function of ROL1. The relative abundance (RA) of these families was altered (BH < 0.05) in *rol1-1* compared to its WT, and the alterations are restored (BH < 0.05) by *ROL1* gene complementation. The Z-scores of family RA are shown in the box plots, *n* = 4 biological replicates. Boxes represent the interquartile range between the first and third quartiles, and the vertical line inside the box defines the median. Whiskers represent the lowest and highest values, respectively. Downward arrows highlight the families whose enrichment was negatively affected by ROL1 dysfunction. (d) ROL1 dysfunction impaired root colonization by *B. megaterium* YC4-R4 and *B. megaterium* TG1-E1 at 13 DAT. Mean ± SE, *n* = 5 biological replicates. (e) ROL1 dysfunction impaired plant growth promotion triggered by *B. megaterium* YC4-R4 and *B. megaterium* TG1-E1 at 13 DAT. Mean ± SE, *n* = 15 biological replicates. Different letters denote significant differences at *P* < 0.05, Tukey's multiple comparison test.

In addition to altering the binary relation between Arabidopsis and GB03, ROL1 dysfunction also reshapes the root-associated microbial community (Fig. 5a and b; Fig. S9). The profiling of Arabidopsis root microbiome from a natural soil identified 11 bacteria families whose association with the root was affected by the function of ROL1 (Fig. 5c; Supplementary Data Set S3), because these families showed altered (BH < 0.05) microbiome enrichment in *rol1-1* compared to wild-type plants, and the alterations were restored by *ROL1* gene complementation. Notably, the enrichment of the *Bacillaceae* family decreased in *rol1-1* (Fig. S10a and b). Consistent with decreased enrichment of *Bacillaceae* in the natural soil, *rol1* plants grown in tyndalized soil showed impaired root colonization of *B. megaterium* YC4-R4 and *B. megaterium* TG1-E1 (Fig. 5d and e), which are two plant-beneficial *Bacillaceae* members [23,24]. Although the complex microbe–microbe and plant–microbe interrelations within the root microbiome are unclear, the decreased enrichment of *Bacillaceae* appears to support the antagonizing effect of ROL1-dependent LDR on plant compatibility with GB03, which belongs to the *Bacillaceae* family.

## DISCUSSION

**Figure 6.**
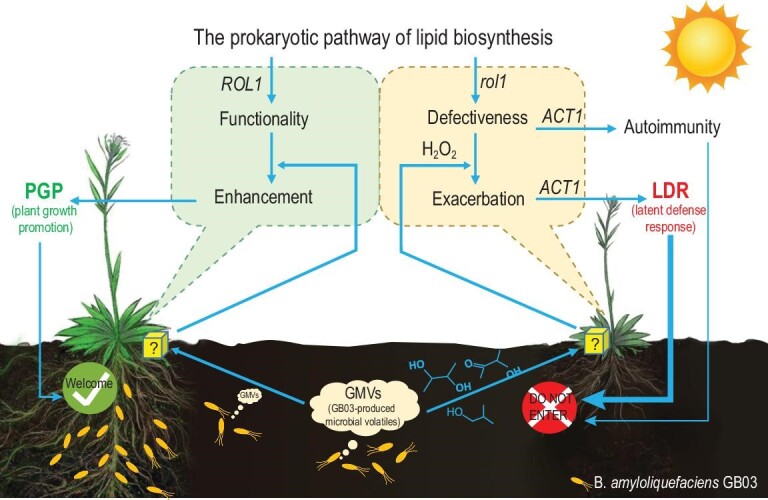
A model of GMV-elicited LDR in the *rol1* mutants. Chloroplastic lipid biosynthesis, which consumes ROL1-dependent 18 : 1, is critical for both GMV-triggered growth promotion and LDR in Arabidopsis. With sufficient 18 : 1, GMV-exposed plants enhance chloroplastic lipid production in supporting bacteria-triggered growth promotion. With deficient 18 : 1, the attempts to enhance chloroplastic lipid production would exacerbate 18 : 1 deficiency, making the association unfavorable for the plant. In *rol1* mutant plants, the unwelcomed association is perceived through GMV-triggered H_2_O_2_ over accumulation, which can drive the oxidation of MGDG and DGDG in priming an SA-dependent defense and increase 18 : 1 consumption in the prokaryotic pathway of chloroplastic lipid biosynthesis. As a result, LDR is conditionally activated to deter the unfavorable plant-rhizobacteria association. The question marks indicate unknown sensors of the bacterial volatiles.

Our forward genetic screening identified ROL1 as an important factor required for plant-rhizobacteria mutualism. More importantly, the loss of plant-rhizobacteria mutualism disclosed the ROL1-regulated LDR, which is mediated through the prokaryotic pathway of chloroplastic lipid biosynthesis (Fig. 6). The conditional activation of LDR avoids unnecessary hostility to compatible microbes while enabling plants to deter the microbial association when it is unfavorable. Therefore, we propose that, parallel to the forefront immunity to MAMPs, an LDR to certain NPFs provides a hidden layer of defense important for controlling compatibility with commensal or beneficial microbes. LDR may commonly exist in various compatible host–microbe combinations and may have evolved to involve both generalized and specialized mechanisms.

Evidence of plant LDR is emerging. In a recent report [17], the GMV component diacetyl (also known as 2,3-butanedione), was shown to induce SA-mediated defense in phosphate (Pi)-deficient plants; whereas in Pi-sufficient plants, diacetyl partially suppresses plant immunity, especially microbial induction of ROS burst. This phenomenon indicates that Pi-deficient plants activate a defense to deter the otherwise (under Pi-sufficient conditions) beneficial rhizobacteria, which compete against the plants for the limited Pi sources in the rhizosphere [17,25,26]. Similarly, the relation between *A. thaliana* and the fungus *Colletotrichum tofieldiae* also showed Pi-dependent transition from mutualism to defense [27], although it remains unclear whether in this case plant defense is triggered by certain non-pathogenic factors. The diacetyl-triggered LDR is mediated through a mechanism different from LDR in *rol1*, because the latter is not induced by diacetyl. Such a difference is consistent with the observation that diacetyl suppresses ROS accumulation whereas LDR in *rol1* requires ROS accumulation.

It remains unclear how the LDR elicitors are perceived by the plants. It is possible that these volatile NPFs are perceived by roots, where certain signals may be generated and transmitted systemically for downstream judgments, for instance, by the chloroplastic lipid biosynthesis pathway to determine potential threat as it accumulates. This scenario would be consistent with the proposed function of LDR in that the need for LDR appears to be not as prompt as the need for the forefront immune responses triggered by MAMPs, since LDR deals with the otherwise beneficial bacteria whereas the MAMP-triggered immunity aims to deter pathogens. The LDR in *rol1* can be activated by 2,3-butanediol, acetoin or 2-methyl-1-propanol. The volatile compounds 2,3-butanediol and acetoin are common to many different strains, more or less randomly, throughout the bacterial kingdom [28]. Therefore, while the activated LDR can antagonize plant compatibility with beneficial strains that produce 2,3-butanediol and acetoin, pathogenic strains that produce these compounds may also activate the LDR, which can then reinforce plant disease resistance in addition to the contribution by the forefront MAMP-triggered immunity.

In this study, a root microbiome was examined to profile the impacts of ROL1 dysfunction on the assembly of root-associated bacteria. The binary association between *B. amyloliquefaciens* GB03 and Arabidopsis is impaired by elevated plant immunity [17]. Similarly, the *rol1*-dependent changes in root microbiome can be attributed, at least partially, to the altered plant immunity, since immunity is a crucial factor that controls plant–microbe interactions [29]. Potential alterations in root exudates may also play an important role in shaping the *rol1* root microbiome, although it is unclear whether such a scenario would be attributed to the alterations in immunity or to the altered lipidome in a way that is independent of immunity, since fatty acids and lipids are important and often essential for various cellular functions beyond defense responses [19,22]. Understanding the potential contributions of root exudates to the altered root–bacteria interactions would require not only *in situ* identification and quantification of root exudates, but also investigations involving genetic and/or biochemical disruptions that precisely mimic the alterations either individually or in combinations.

Difficulties exist in explicitly understanding the bacteria species diversity within the microbiome. For instance, some bacterial species are increased in the *rol1* root microbiome; although this appears to be inconsistent with the elevated plant immunity, such a pattern reflects a balanced outcome of the complex interactive network of plant–microbe and microbe–microbe interactions, yet it remains challenging to understand, at the community level, why and how each microbiome member ends up with the observed patterns. Despite the limitations, our microbiome profiling revealed an interesting pattern, i.e. the *rol1* microbiome showed decreased relative abundance of the *Bacillaceae* family, which is known to contain many plant-beneficial strains including *B. amyloliquefaciens* GB03 [4,30]. Although a coincidence cannot be ruled out, the decrease of *Bacillaceae* in the *rol1* root microbiome is consistent with the observation that GB03 colonization is reduced in *rol1*, and thus appears to support the importance of ROL1 for plant compatibility with beneficial bacteria.

Wild-type plants benefit from the association with GB03 via multiple mechanisms, including the fact that GMVs increase the levels of major chloroplastic lipids. In contrast, *rol1* plants are at risk from the association with GB03 because the GMV-induced attempts to enhance chloroplastic lipid production would threaten to exacerbate the 18 : 1 deficiency and consequent disruptions in the plant lipidome. Therefore, although the GMV treatment still promotes *rol1* mutant growth to a certain degree, the association with GB03 is actually risky and can be costly for *rol1*, since fatty acids and lipids are important and often essential for various cellular functions [19,22]. In this sense, LDR reflects not only the plant's vigilance to potential threats from compatible microbes, but also the plant's ability to control compatibility with certain beneficial microbes.

## MATERIALS AND METHODS

The *rol1-1* mutant allele was isolated from a forward genetic screening of an EMS mutant pool in this study. The *rol1-2* (SAIL-209_D07), *act1-5* (SALK_069657) and *eds1* (SALK_057149) mutants were ordered from the NASC (Nottingham Arabidopsis Stock Centre) or ABRC (Arabidopsis Biological Resource Center). *NahG* was from Prof. Alberto Macho at the Shanghai Center for Plant Stress Biology (PSC). The double mutants of *act1-5 rol1-1*, *eds1 rol1-1* and *NahG rol1-1* were generated by genetic cross. The *srfr1-4* (Sail_412_E08) and *bon1-3* (SALK_200380) were provided by Prof. Yang Zhao at PSC. Details about plant growth conditions are described in the Materials and Methods section of the Supplementary Materials, which includes the following subsections: Bacteria growth and inoculum preparation; Plant growth promotion by bacteria inoculation in soil; Plant growth promotion by GMV exposure in plates; EMS mutant screening and map-based cloning; Gene complementation; Chemical treatments for LDR tests; RNA seq and analysis; Quantitative real-time PCR; MAP kinase assay; Lipidome measurements and data analysis; Hydrogen peroxide treatment, scavenging and staining; Microbiome sample preparation and 16 s rRNA gene sequencing; Microbiome data analysis; and Measurements of root-colonized bacteria.

## Supplementary Material

nwac109_Supplemental_Files
